# Algicidal Activity of Bacillamide Alkaloids and Their Analogues against Marine and Freshwater Harmful Algae

**DOI:** 10.3390/md15080247

**Published:** 2017-08-07

**Authors:** Bo Wang, Yuanyuan Tao, Qisheng Liu, Na Liu, Zhong Jin, Xiaohua Xu

**Affiliations:** 1 State Key Laboratory of Elementoorganic Chemistry, Nankai University, Tianjin 300071, China; wangbonankai@126.com (B.W.); yuanyuantaochem@mail.nankai.edu.cn (Y.T.); liuqisheng@mail.nankai.edu.cn (Q.L.); xielongguan@nankai.edu.cn (N.L.); zjin@nankai.edu.cn (Z.J.); 2 Collaborative Innovation Center of Chemical Science and Engineering (Tianjin), Tianjin 300071, China

**Keywords:** algicide, bacillamide, thiazolamide, alleopathy

## Abstract

Harmful algal blooms have become a great challenge to global aquatic ecosystems over the past decades. Given their low toxicity, high selectivity, and environment-friendly properties, the use of natural products and their analogues as algicides has proven to be particularly efficient. In the present study, algicidal activity of naturally occurring bacillamides A–C, alkaloid (**1**), and neobacillamide A, as well as their synthetic analogues were investigated intensively. Bioassay results showed that, relative to natural bacillamide alkaloids, aniline-derived analogue (**10d**) exhibited higher algicidal potential against three freshwater harmful algae *Mycrocyctis aeruginosa*, *Scenedesmus obliquus*, and *Chlorella pyrenoidosa*, suggesting that it could be used as a promising lead compound to develop novel algicide for controlling harmful algal blooms.

## 1. Introduction

Currently, harmful algal blooms (HABs) are emerging as an ever-increasing environmental problem, which do not only influence greatly aquatic-ecosystem sustainability and water resource conservation, but also pose a direct threat to human and animal health as well as economic growth worldwide [1,2,3]. To date, a large number of strategies, which are basically classified into three types: (1) physical methods, e.g., clays, surfactants, and flocculants [4,5,6]; (2) chemical methods, e.g., copper sulfate, sodium hypochlorite, and herbicides such as Diuron, Endothal, Atrazine, and so on [7,8,9,10]; and (3) biological methods, e.g., algicidal bacteria, viruses, and plankton grazers [11,12,13], have been developed for controlling HABs. In spite of these significant advances, unfortunately, all these methods are either too expensive to implement or nonspecific to harmful algae. Therefore, developing inexpensive, selective and eco-friendly algicidal agents against harmful algae still remains a great challenge and active area of research.

**Figure 1:**
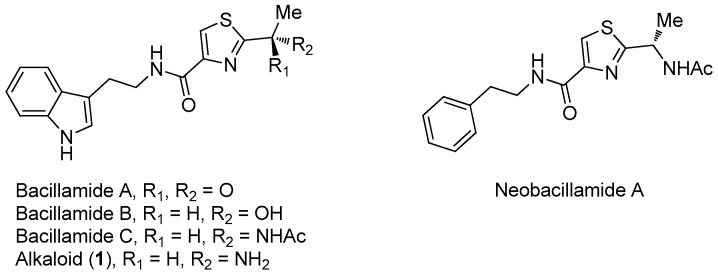
Bacillamide alkaloids.

Recently, intensive efforts on the use of natural products (NPs) and bacteria to control HABs have been made [14]. Several examples involving the application of compounds derived from natural products as efficient algicidal agents have been reported over the past two decades [15,16,17,18,19,20,21]. Meanwhile, algicidal activity of bacteria was usually thought to be highly associated with chemicals secreted by bacteria [22,23,24,25,26,27]. From the viewpoints of chemical ecology, interaction between algae and bacteria that release algicidal secondary metabolites, namely alleopathy [28], possesses inherent merits such as low mammalian toxicity, high selectivity, easy degradability, and environment-friendly properties. As a result, much interest has been focused on the use of bacteria for controlling blooms of harmful algae in marine and freshwater environments. Some of the related algicidal chemicals have been isolated and identified from these bacteria [29,30,31,32,33,34,35,36]. Amongst them, bacillamide alkaloids, produced by marine *Bacillus* sp. in succession (Figure 1) [37], contain a common substituted thiazole tryptamide motif. As the first reported secondary metabolite of this family, alkaloid (**1**), *N*-(2-(1*H*-indol-3-yl)ethyl)-2-acetylthiazole-4-carboxamide, was originally isolated from freshwater *Thermoactinomyces* strain TM-64 in 1976 [38]. Recently, a new member of this family, neobacillamide A, possessing a unique thiazole phenylethylamide moiety in the molecular skeleton, was isolated from marine bacterium *Bacillus vallismortis* [39]. Bacillamide A, first isolated from a marine *Bacillus endophyticus* collected during termination of a toxic algal bloom [40], was shown to display antibiotic activity against dinoflagellates and raphidophytes. Unfortunately, due to the insufficient availability of alkaloids of this class from nature, there are not many more algicidal activities that have been reported to date [41].

In our previous work [42], we have described an efficient synthesis of bacillamides A–C. Herein, we reported algicidal activity of alkaloids of this class against several marine and freshwater harmful algae. In addition, several analogues of bacillamides A and B were also prepared and evaluated for their algicidal activities in order to investigate their structure-activity relationship.

## 2. Results

### 2.1. Synthesis of Bacillamide Alkaloids

**Scheme 1:**
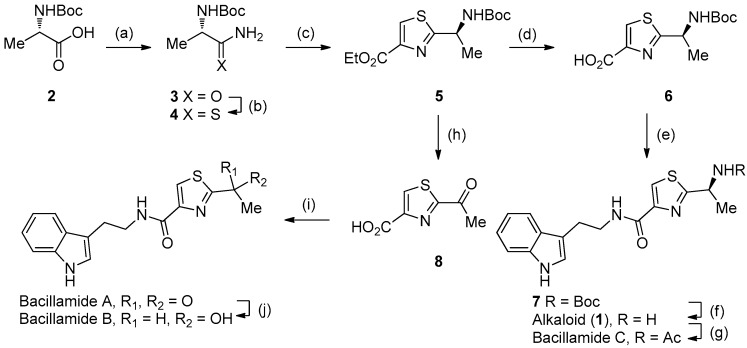
Synthesis of bacillamide alkalolids (Adapted from [42]. Copyright Shanghai Institute of Organic Chemistry and Chinese Academy of Sciences, 2010.)

Recently, we have successfully achieved a total synthesis of bacillamides A–C utilizing readily available L-alanine as the starting material (Scheme 1) [42]. Firstly, *N*-Boc L-alanine (**2**) was smoothly converted into the amide (**3**) in excellent yield. Subsequently, the thioamide (**4**) was produced in the presence of P_2_S_5_. Cyclization of thioamide with ethyl 3-bromopyruvate proceeded successfully to afford the key intermediate thiazole carboxylate ester (**5**) in good yield. Hydrolysis of ethyl ester under basic conditions afforded the corresponding carboxylic acid (**6**) followed by amidation with tryptamine to give the *N*-Boc protected amide (**7**) in high overall yield. After removal of *N*-Boc protection group, the desired alkaloid (**1**) was obtained in excellent yield. Finally, selective *N*-acetylation with Ac_2_O in the presence of pyridine gave alkaloid bacillamide C in excellent yield. Alternatively, ester (**5**) could also be converted into the carboxylic acid (**8**) via oxidation of amine followed by hydrolysis. The amidation of acid (**8**) successfully afforded alkaloid bacillamide A, which was smoothly reduced to give bacillamide B in almost quantitative yield.

**Scheme 2:**
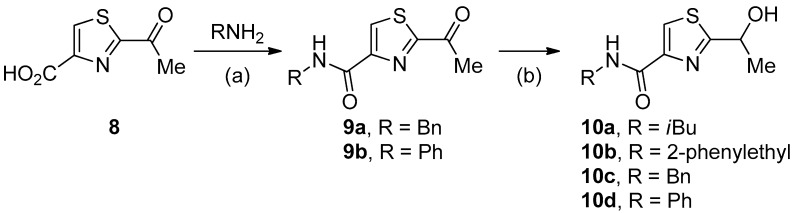
Preparation of amide analogues of bacillamides A and B. Reagents and conditions: (a) isobutyl chloroformate, NMM, CH_2_Cl_2_, then amine, NMM, CH_2_Cl_2_, room temperature; (b) NaBH_4_, MeOH/CHCl_3_, room temperature.

In a similar manner, four amide analogues (**10a**–**d**) of bacillamide B and two analogues (**9a** and **9b**) of bacillamide A were readily prepared from the intermediate thiazole carboxylic acid (**8**) in good yields (Scheme 2).

### 2.2. Algicidal Activity

Natural bacillamide alkaloids A–C, alkaloid (**1**), and neobacillamide A, as well as their synthetic analogues (**9a**, **9b**, and **10a**–**d**) were evaluated for their algicidal activity against two typical red tide algae *Skeletonema costatum* and *Gymnodinium catenatum*, and three freshwater harmful algae *Mycrocyctis aeruginosa*, *Scenedesmus obliquus*, and *Chlorella pyrenoidosa*. The bioassay results were outlined in Table 1.

## 3. Discussion

### 3.1. Synthesis

Synthetically, bacillamide C has been synthesized in a racemic form by Wang et al. [43]. The key transformation involves a thiazole Ugi multicomponent cyclization reaction. At almost the same time, we also described the first enantioselective synthesis of bacillamide C and alkaloid (**1**) using L-alanine as the starting material [42]. In addition, bacillamides A and B could also be produced by utilizing the thiazole carboxylic acid (**8**) as the key intermediate. The versatility of this synthetic strategy was further demonstrated in preparation of several amide analogues of bacillamides A and B.

### 3.2. Algicidal Activity

Algicidal activity for natural bacillamide alkaloids and their synthetic analogues against marine and freshwater harmful algae were listed in Table 1.

From data outlined in Table 1, natural bacillamide alkaloids exhibited good algicidal effects against both red tide algae. Amongst them, bacillamide A displayed excellent algicidal efficacy against *S. costatum* with EC_50_ value as low as 0.011 mg/L, while alkaloid (**1**) showed potent algicidal activity (EC_50_ = 0.58 mg/L) against *G. catenatum*, which produces the toxic brevetoxin when they form a red tide bloom. In contrast, both bacillamides B and C revealed moderate algicidal effects against *S. costatum* and *G. catenatum*.

Cyanobacterial blooms have become a worldwide aquatic environmental problem because of ever-increasing water eutrophication and climate change. The most popular harmful algal blooming species found in freshwater belongs to the cyanobacteria *M. aeruginosa* and *S. obliquus*. As shown in Table 1, natural bacillamides generally showed moderate algicidal potential against three freshwater harmful algae. Comparably, bacillamide A exhibited highest algicidal efficacy (EC_50_ = 19.3 mg/L) against *M. aeruginosa*, whereas neobacillamide A and alkaloid (**1**) showed potent inhibitory activity against *S. obliquus* with an EC_50_ value of 5.0 and 14.3 mg/L, respectively.

Encouragingly, all amide analogues (**10a**–**d**) of bacillamide B exhibited higher algicidal activity against three freshwater harmful algae than parent bacillamide B. Based on the significant difference of the aldicidal activity between compounds (**10a**–**d**) and natural bacillamide B, it was therefore inferred that, not tryptamide motif, but thiazole amide scaffold turned out to be the active pharmacophore for the algicidal activity. Relative to alkyl amine-derived analogues (**10a**–**c**), aniline-derived analogue (**10d**) was found to show highest algicidal potential against three harmful algae with an EC_50_ value of 2.5, 4.0, and 23.0 mg/L, respectively, suggesting that this analogue could be used as a lead compound for further development of algicides to control HABs. In addition, amide analogue (**9b**) of bacillamide A also exhibited comparable algicidal activity with that of the corresponding precursor alcohol (**10d**) against *M. aeruginosa* and *S. obliquus*.

## 4. Materials and Methods

Melting points were determined using an X-4 digital melting point apparatus (Taike HighTech Co., Beijing, China) and thermometer uncorrected. ^1^H and ^13^C nuclear magnetic resonanance (NMR) spectra were recorded in deuterochloroform or deutero dimethyl sulfoxide solution with a BRUKER (400 MHz) instrument (Rheinstetten, Germany) using tetramethylsilane as an internal standard, and chemical shift values (δ) were given in parts per million. High resolution mass spectrometer (HRMS) data were obtained on a high resolution ESI-FTICR mass spectrometer (Ionspec 7.0 T, Varian Inc., Palo Alto, CA, USA). All reagents were commercially available and treated according to the standard methods prior to use. Bacillamides A–C, alkaloid (**1**), and neobacillamide A were prepared according to our previous work [42].

### 4.1. General Procedure for Synthesis of Analogues of Bacillamide A

To a cooled (0 °C) solution of the carboxylic acid **8** (0.81 g, 4.73 mmol) in dichloromethylene (100 mL) was added *N*-methyl morpholine (0.5 mL, 4.73 mmol) and *iso*-butyl chloroformate (0.6 mL, 4.73 mmol). The mixture was stirred at 0 °C for 1 h. The resultant mixture was then added into a solution of amine (4.26 mmol) and *N*-methyl morpholine (0.5 mL, 4.73 mmol) in dichloromethylene (20 mL) and the reaction mixture was stirred at room temperature for another 2 h. Water (30 mL) was added to the mixture and the layers were separated. The aqueous phase was extracted with dichloromethylene (20 mL). The combined organic phase was dried over anhydrous MgSO_4_. After removal of solvent under reduced pressure, the residue was purified by silica gel column chromatography (PE/EA = 1:1, *v*/*v*) to give the corresponding amide **9**.

### 4.2. General Procedure for Synthesis of Analogues of Bacillamide B

To a solution of compound **9** (3.39 mmol) in MeOH/CHCl_3_ (140 mL, *v/v* = 2:3) was added NaBH_4_ (155 mg, 4.07 mmol, 1.2 equiv) in small portions at 0 °C. After completion of addition, the mixture was stirred for another 2 h and monitored by TLC. The solvent was removed by vacuum and the residue was further purified by silica gel column chromatography (PE/EA = 1:1, *v/v*) to give the desired products **10**.

The characteristic data for these derivatives were outlined as the following:

*2-Acetyl-N-benzylthiazole-4-carboxamide* (**9a**). Yellow solid, m.p. 95–97 °C. ^1^H NMR (Figure S1) (400 MHz, CDCl_3_) δ 8.46 (s, 1H), 7.69 (s, 1H), 7.46–7.22 (m, 5H), 4.69 (d, *J* = 5.8 Hz, 2H), 2.69 (s, 3H). ^13^C NMR (Figure S2) (101 MHz, CDCl_3_) δ 191.1, 167.0, 160.6, 151.6 138.1, 130.0, 128.8, 127.9, 43.4, 26.0. HRMS (ESI) calcd. for. C_13_H_12_N_2_O_2_S [M + H]^+^: 261.0691, found: 261.0687.

*2-Acetyl-N-phenyl-thiazole-4-carboxamide* (**9b**). Yellow solid, m.p. 134–136 °C. ^1^H NMR (Figure S3) (400 MHz, CDCl_3_) δ 8.99 (s, 1H), 8.44 (s, 1H), 7.66 (d, *J* = 8.1 Hz, 2H), 7.33–7.31 (m, 2H), 7.11 (m, 1H), 2.72 (s, 3H). ^13^C NMR (Figure S4) (101 MHz, CDCl_3_) δ 189.9, 165.7, 157.1, 150.7, 136.2, 129.4, 128.2, 123.8, 119.0, 25.0. HRMS (ESI) calcd. for. C_12_H_10_N_2_O_2_S [M + H]^+^: 247.0463, found: 247.0983.

*(S)-2-(1-Hydroxyethyl)-N-iso-butylthiazole-4-carboxamide* (**10a**). Yellow oil. ^1^H NMR (Figure S5) (400 MHz, *d*_6_-DMSO) δ 8.23 (t, *J* = 5.8 Hz, 1H), 8.13 (s, 1H), 6.25 (d, *J* = 4.9 Hz, 1H), 5.05–4.88 (m, 1H), 3.09 (dd, *J* = 12.9, 6.6 Hz, 2H), 1.85 (dt, *J* = 13.5, 6.7 Hz, 1H), 1.49 (d, *J* = 6.5 Hz, 3H), 0.86 (d, *J* = 6.7 Hz, 6H). ^13^C NMR (Figure S6) (101 MHz, *d*_6_-DMSO) δ 178.5, 160.6, 149.8, 122.8, 66.6, 28.1, 24.1, 20.0. ESI-HRMS: calcd. for C_10_H_16_N_2_O_2_S [M + H]^+^, 229.0932, found: 229.1012.

*(S)-2-(1-Hydroxyethyl)-N-phenylethylthiazole-4-carboxamide* (**10b**). Pale yellow oil. ^1^H NMR (Figure S7) (400 MHz, *d*_6_-DMSO) δ 8.33 (s, 1H), 8.14 (s, 1H), 7.43–7.12 (m, 5H), 6.28 (s, 1H), 5.04–4.82 (m, 1H), 3.49 (d, *J* = 6.2 Hz, 2H), 2.85 (t, *J* = 6.8 Hz, 2H), 1.49 (d, *J* = 5.6 Hz, 3H). ^13^C NMR (Figure S8) (101 MHz, *d*_6_-DMSO) δ 178.6, 160.5, 149.7, 139.3, 128.5, 128.3, 126.1, 123.0, 66.6, 40.2, 40.1, 24.1. ESI-HRMS: calcd. for C_14_H_16_N_2_O_2_S [M + H]^+^, 277.0932, found: 277.1011.

*(S)-N-Benzyl-2-(1-hydroxyethyl)thiazole-4-carboxamide* (**10c**). Pale yellow oil. ^1^H NMR (Figure S9) (400 MHz, *d*_6_-DMSO) δ 8.86 (s, 1H), 8.18 (s, 1H), 7.32 (s, 4H), 7.24 (s, 1H), 4.97 (d, *J* = 5.9 Hz, 1H), 4.46 (d, *J* = 4.7 Hz, 2H), 1.50 (d, *J* = 5.7 Hz, 3H). ^13^C NMR (Figure S10) (101 MHz, *d*_6_-DMSO) δ 178.7, 160.6, 149.6, 139.6, 128.2, 127.3, 126.7, 123.3, 66.6, 42.1, 24.1. ESI-HRMS: calcd. for C_13_H_14_N_2_O_2_S [M + H]^+^, 263.0776, found: 263.0853.

*(S)-2-(1-Hydroxyethyl)-N-phenylthiazole-4-carboxamide* (**10d**). Yellow oil. ^1^H NMR (Figure S11) (400 MHz, *d*_6_-DMSO) δ 10.09 (s, 1H), 8.35 (s, 1H), 7.83 (d, *J* = 7.3 Hz, 2H), 7.36 (d, *J* = 6.6 Hz, 2H), 7.12 (s, 1H), 6.34 (s, 1H), 5.05 (s, 1H), 1.55 (d, *J* = 4.8 Hz, 3H). ^13^C NMR (Figure S12) (101 MHz, *d*_6_-DMSO) δ 178.9, 159.2, 149.5, 138.3, 128.5, 124.5, 123.8, 120.4, 66.7, 24.2. ESI-HRMS: calcd. for C_12_H_12_N_2_O_2_S [M + H]^+^, 249.0619, found: 249.0697.

### 4.3. Algicide Bioassay

#### 4.3.1. Bioassay Method for Red Tide Algae

Two typical red tide algae *S. costatum* and *G. catenatum* were selected to perform exogenous substance exposure experiments. The cultures of algae *S. costatum* and *G. catenatum* were obtained from Ocean University of China, Qingdao, China. Algal culture conditions: seawater medium (f/2), lighting ration (14 h/10 h), light intensity during the day (12,000 Lux), and culture temperature (20–25 °C).

A preliminary experiment was first carried out to determine the highest inhibitory concentration which acetone as the solvent has no obvious effect on algae. Drug agent was dissolved in acetone and added into algae cultivation system in logarithmic growing period. The algae without drug agent were used as the control experiments. Each bioassay was repeated for three times at every concentration. The measurement of algal cell density was observed by Hemocytometer under a microscope (Nikon YS100, Nikon Instruments, Tokyo, Japan).

Data processing and evaluation method: Half inhibitory concentration (EC_50_ value) for each drug after 72 h was calculated based on the inhibitory ratio (*IR*%) of drug agent against each algae. The inhibitory ratio for each drug was determined according to the following equation:

*IR* (%) = (1 − *N*/*N*_0_) × 100%

where *N* is algal density of test sample (with drug agent), *N*_0_ is algal density of the control experiment (without drug). The standard deviation was calculated and data reported as the mean ± SD.

#### 4.3.2. Bioassay Method for Freshwater Harmful Algae

Three freshwater algae species *Mycrocyctis aeruginosa*, *Scenedesmus obliquus* and *Chlorella pyrenoidosa* were used to establish bioassay method. (Note: Cyanobacteria *M. aeruginosa* is most popular in freshwater bodies in China.) Three algae species were obtained from the Freshwater Algae Culture Collection of the Institute of Hydrobiology (FACHB-collection). The culture conditions used were similar to Schrader’s method [44], except that BG-11 and SE were used as the positive media for three algae, and pH value was maintained at 7.1–7.2. The algae were preserved in continuous steady-state cultures to provide a source of cells growing at a fairly constant rate. The algae were maintained at 25 °C with alternating light at 4000 lux.

Bacillamides and their analogs were evaluated using a microplate method in the laboratory and bioassay was performed as described by Schrader et al. [17]. Bacillamides and their analogs were separately dissolved in DMSO to produce the corresponding stock solutions. The resulting solutions were pipetted into each bottle of algal cultures, which had been incubated at continuous conditions for 4 days. At that time, the algal cultures were at the exponential growth phase and ideal for evaluation of algicidal activity. The volume of the medium and algae were calculated using the equation: *Y* = (5.5 × 10^5^ × 40)/*X* (*X*, the algal density; *Y*, the volume of algae). A 5 mL aliquot of the respective algal culture was pipetted into 35 mL of the medium as the initial algal concentration. The concentrations of the natural products were 300, 200, 100, 50, 25, 12.5, 6.25, 3.12, and 1.56 ppm in the bottles after 20 µL of the respective stock solution was pipetted into each bottle. Three replicates of each natural product at a given concentration as well as the controls were prepared. These bottles were incubated in continuous conditions for another 4 days. The optical densities of the test wells were measured at 650 nm for *C. pyrenoidosa* and *M. aeruginosa* and at 670 nm for *S. obliquus*. The mean values of the optical density measurements for each treatment concentration and the controls were plotted; these graphs were used to determine the EC_50_ value for each natural product (EC_50_ is the concentration which 50% of algae was inhibited).

On the basis of area values under the individual growth curves, the inhibitory ratio (%) was determined by individual tested concentration according to the following equation:

*I_i_* = (*A_c_* − *A_i_*)/*A_c_* × 100%

where *I_i_* is inhibitory ratio (%) of biomass growth by toxicant concentration *i* (%), *A_c_*: average area under the growth curve by control, *A_i_*: average area under the growth curve by tested concentration *i.*

Mean EC_50_ value of each compound after 96 h was determined for each group of plates. The standard deviation was calculated and data reported as the mean ± SD. Using obtained data on inhibition calculate the toxicity index EC_50_ with appropriate 95% confidence interval based on non-linear regression.

## 5. Conclusions

In summary, the algicidal activity of natural bacillamide alkaloids and their synthetic analogues against several marine and freshwater harmful algae was investigated. The bioassay results showed that some derivatives displayed higher algicidal activity than natural alkaloids, suggesting that they could be used as lead compounds to develop novel algicides for controlling HABs. The preliminary structure-activity relationship studies revealed that, instead of tryptamide motif, thiazole amide scaffold was the active pharmacophore for algicidal activity. Further studies on their environmental safety in catfish ponds, toxicity toward non-target organisms, and potential accumulation in the flesh of channel catfish, as well as algicidal mechanisms, are being pursued in this laboratory.

## Figures and Tables

**Table 1:** Algicidal activity of bacillamide alkaloids and their synthetic analogues ^1^.

Compound	EC_50_ (mg/L) after 72 h		EC_50_ (mg/L) after 96 h		
	*S. costatum*	*G. catenatum*	*M. aeruginosa*	*S. obliquus*	*C. pyrenoidosa*
Bacillamide A	0.011 ± 0.002	112.0 ± 2.58	19.33 ± 1.45	43.76 ± 3.67	250.10 ± 4.56
Bacillamide B	15.59 ± 1.12	234.56 ± 7.96	75.62 ± 5.34	219.38 ± 13.56	387.77 ± 10.78
Bacillamide C	34.70 ± 1.13	57.13 ± 2.54	291.92 ± 10.32	29.54 ± 1.54	192.83 ± 6.95
Alkaloid (**1**)	8.65 ± 0.78	0.58 ± 0.06	114.60 ± 3.35	14.33 ± 1.15	381.57 ± 5.16
Neobacillamide A	-	-	86.90 ± 5.45	4.71 ± 0.46	57.87 ± 3.12
**10a**	-	-	13.1 ± 1.54	26.7 ± 2.37	32.1 ± 1.57
**10b**	-	-	32.1 ± 2.12	12.2 ± 0.36	23.6 ± 2.18
**10c**	-	-	34.0 ± 3.13	11.6 ± 0.98	33.9 ± 2.65
**10d**	-	-	2.5 ± 0.12	4.0 ± 0.43	23.0 ± 1.28
**9a**	**-**	**-**	7.56 ± 0.21	13.12 ± 0.56	7.81 ± 0.65
**9b**	**-**	**-**	2.13 ± 0.02	3.25 ± 0.14	28.91 ± 1.23

^1^ EC_50_ value is the concentration when the growth of half of the algae was inhibited.

## Supplementary Materials

The following are available online at www.mdpi.com/1660-3397/15/8/247/s1, NMR spectra data for new compounds. Figure S1. ^1^H NMR of Compound **9a** (CDCl_3_); Figure S2. ^13^C NMR of Compound **9a** (CDCl_3_); Figure S3. ^1^H NMR of Compound **9b** (CDCl_3_); Figure S4. ^13^C NMR of Compound **9b** (CDCl_3_); Figure S5. ^1^H NMR of Compound **10a** (*d_6_*-DMSO); Figure S6. ^13^C NMR of Compound **10a** (*d_6_*-DMSO); Figure S7. ^1^H NMR of Compound **10b** (*d_6_*-DMSO); Figure S8. ^13^C NMR of Compound **10b** (*d_6_*-DMSO); Figure S9. ^1^H NMR of Compound **10c** (*d_6_*-DMSO); Figure S10. ^13^C NMR of Compound **10c** (*d_6_*-DMSO); Figure S11. ^1^H NMR of Compound **10d** (*d_6_*-DMSO); Figure S12. ^13^C NMR of Compound **10d** (*d_6_*-DMSO).
