# Supplementary material for: Algicidal Activity of Bacillamide Alkaloids and Their Analogues against Marine and Freshwater Harmful Algae
Source: Mar Drugs. 2017 Aug 7;15(8):247. doi: 10.3390/md15080247 (PMC5577602; doi:10.3390/md15080247)
Supplement: Supplementary file 1 [file marinedrugs-15-00247-s001.pdf]

Supplementary Materials: Algicidal Activity of Bacillamide Alkaloids and Their Analogues against Marine and Freshwater Harmful Algae

Bo Wang , Yuanyuan Tao , Qisheng Liu , Na Liu , Zhong Jin and Xiaohua Xu

Contents

Figure S1. ^{1}H NMR of Compound **9a** ($CDCl_3$)..2

Figure S2. ^{13}C NMR of Compound **9a** ($CDCl_3$)..2

Figure S3. ^{1}H NMR of Compound **9b** ($CDCl_3$)..3

Figure S4. ^{13}C NMR of Compound **9b** ($CDCl_3$)..3

Figure S5. ^{1}H NMR of Compound **10a** (*d_6*-DMSO)..4

Figure S6. ^{13}C NMR of Compound **10a** (*d_6*-DMSO)..4

Figure S7. ^{1}H NMR of Compound **10b** (*d_6*-DMSO)..5

Figure S8. ^{13}C NMR of Compound **10b** (*d_6*-DMSO)..5

Figure S9. ^{1}H NMR of Compound **10c** (*d_6*-DMSO)..6

Figure S10. ^{13}C NMR of Compound **10c** (*d_6*-DMSO)..6

Figure S11. ^{1}H NMR of Compound **10d** (*d_6*-DMSO)..7

Figure S12. ^{13}C NMR of Compound **10d** (*d_6*-DMSO)..7

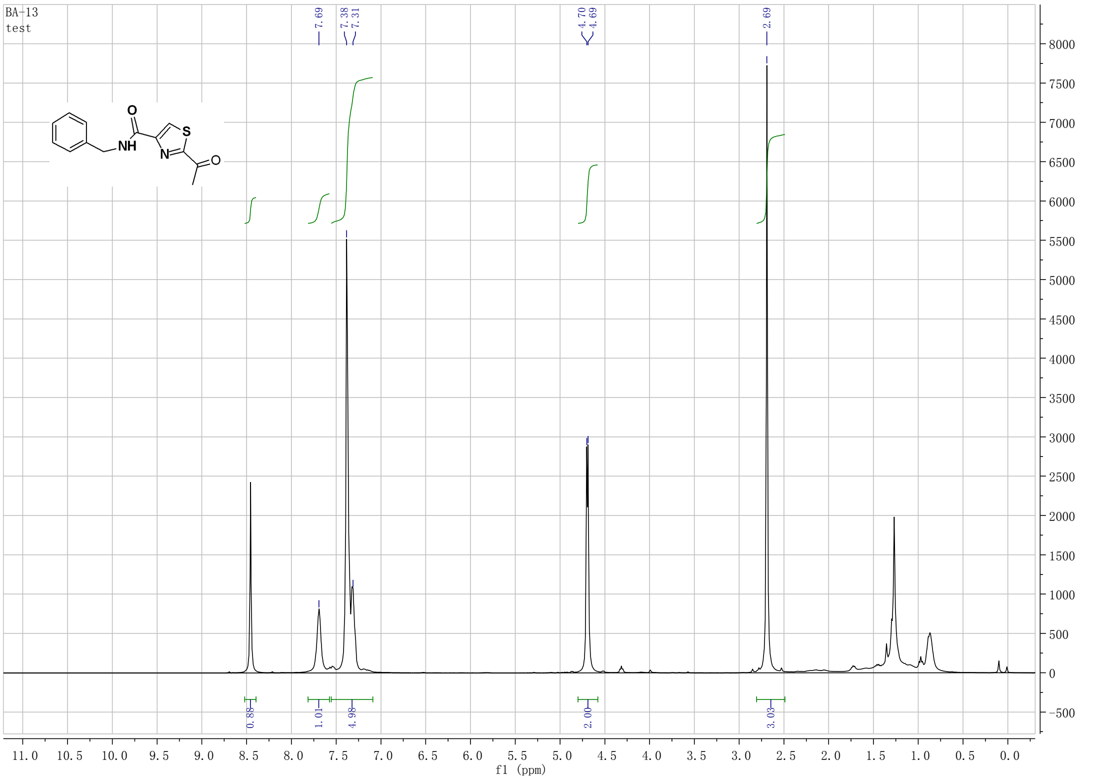

Figure S1. ^{1}H NMR of Compound **9a** ($CDCl_3$).

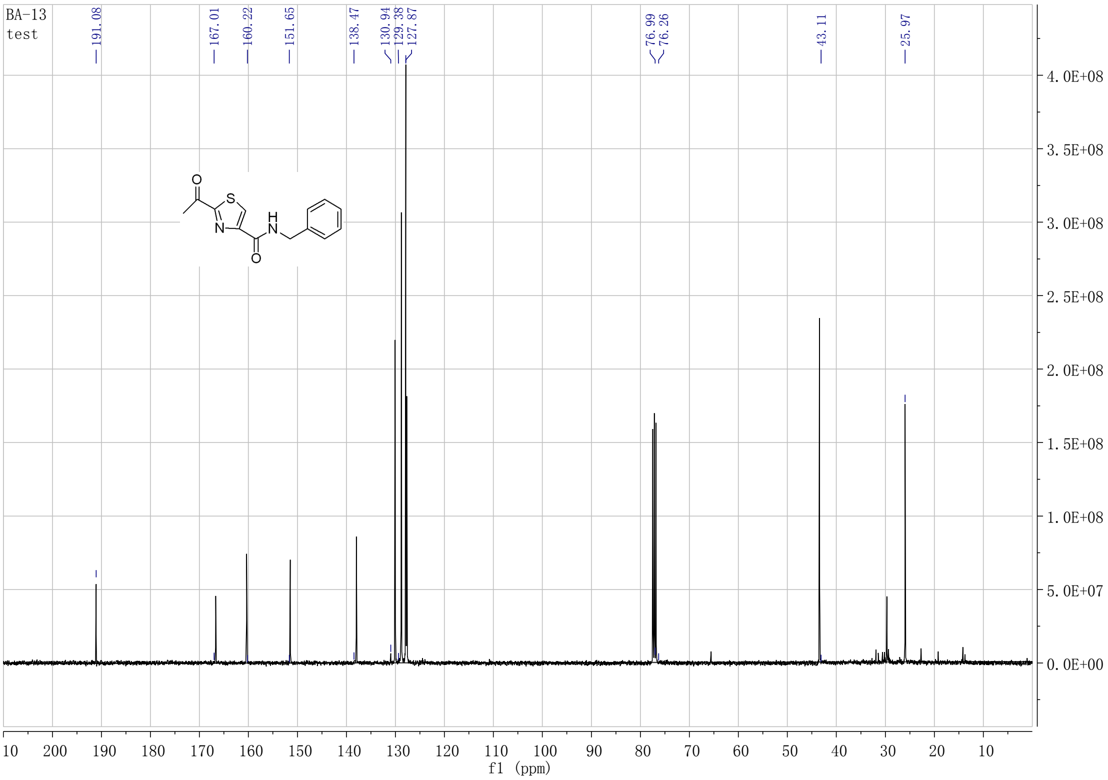

Figure S2. ^{13}C NMR of Compound **9a** ($CDCl_3$).

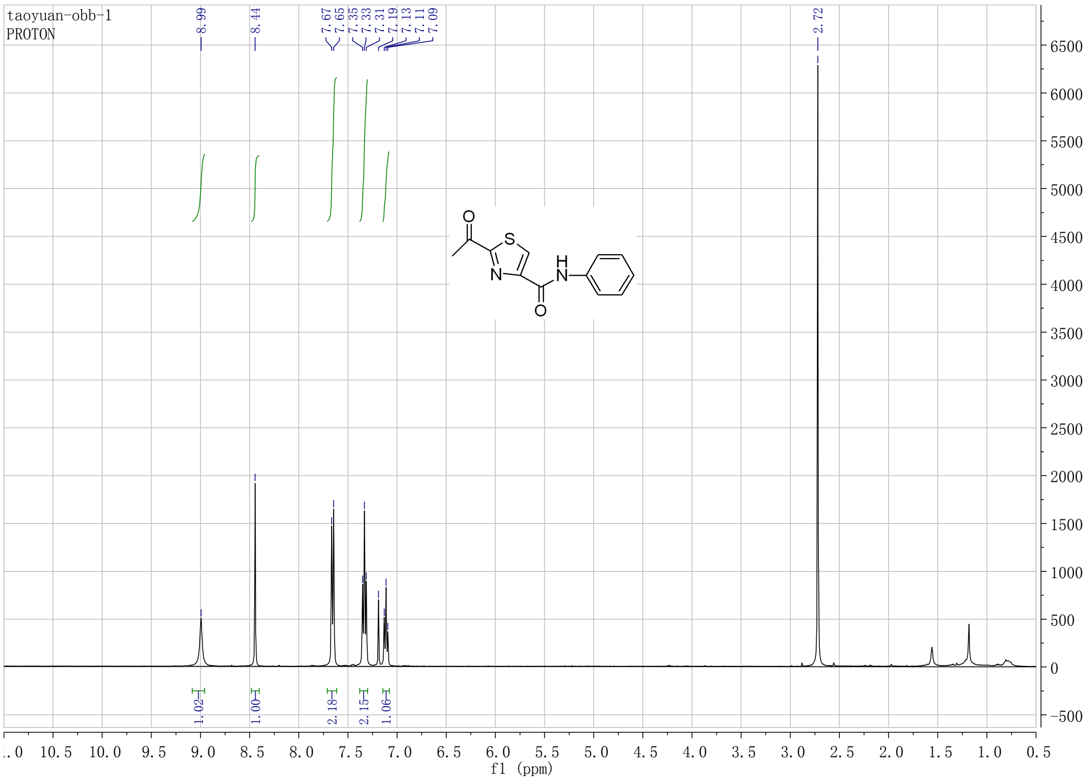

Figure S3. ^{1}H NMR of Compound **9b** ($CDCl_3$).

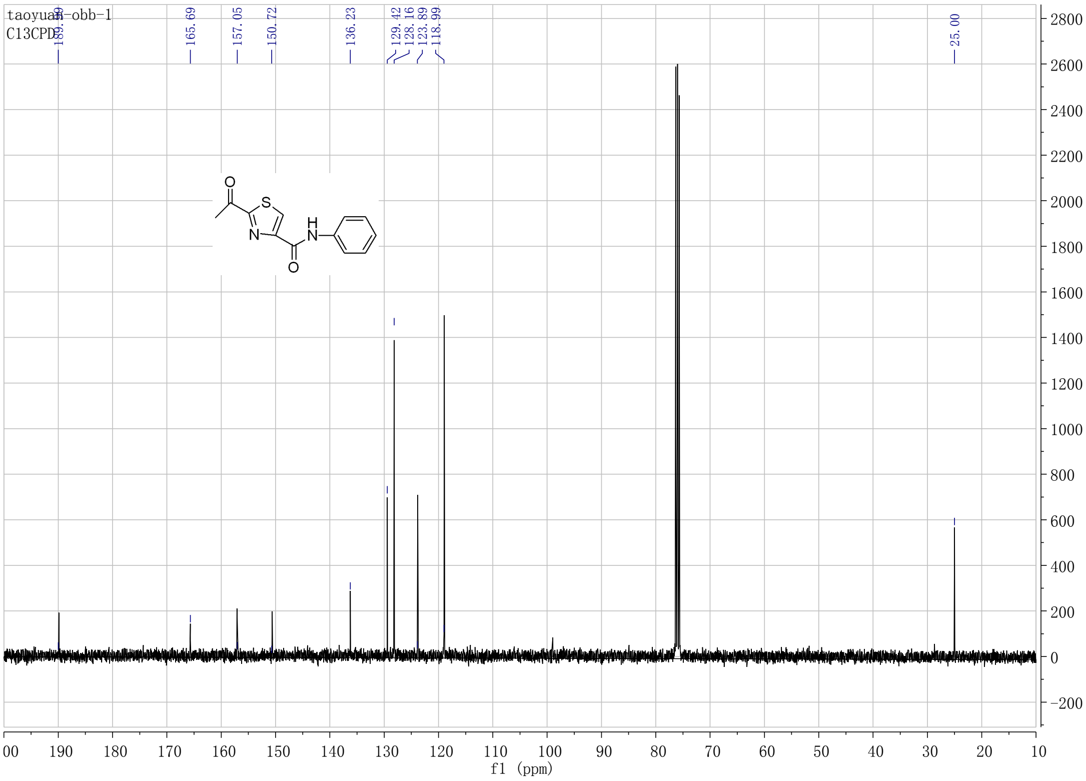

Figure S4. ^{13}C NMR of Compound **9b** ($CDCl_3$).

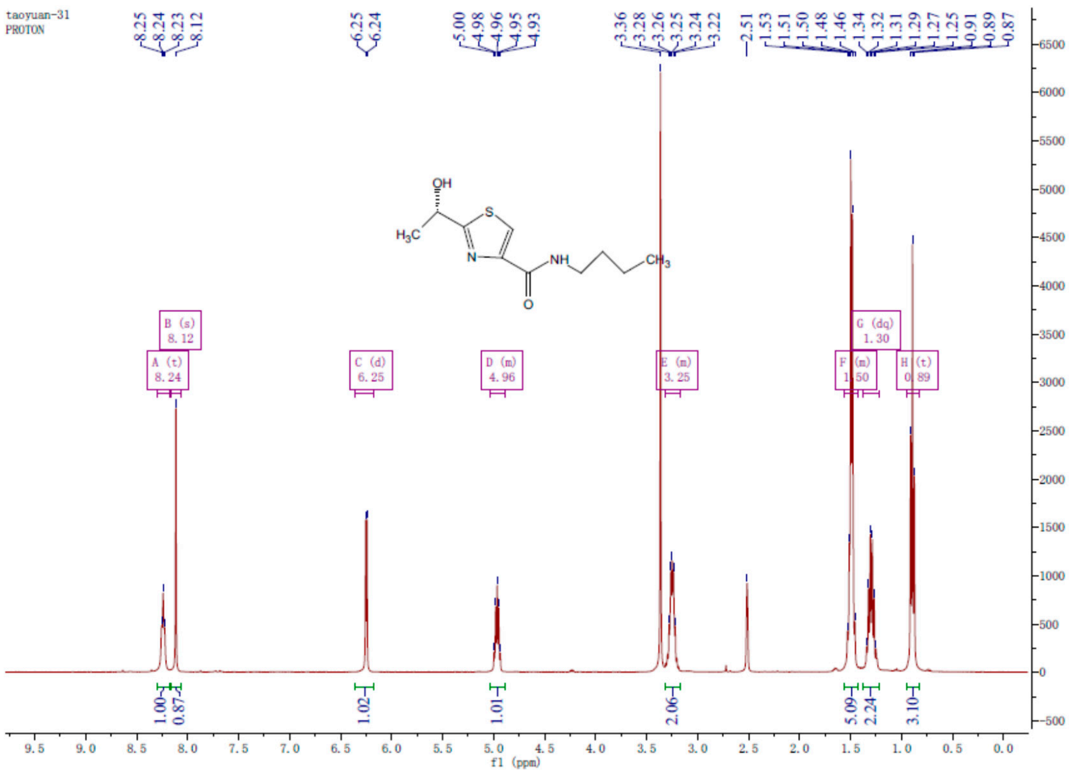

Figure S5. ^{1}H NMR of Compound **10a** (*d*$_6$-DMSO).

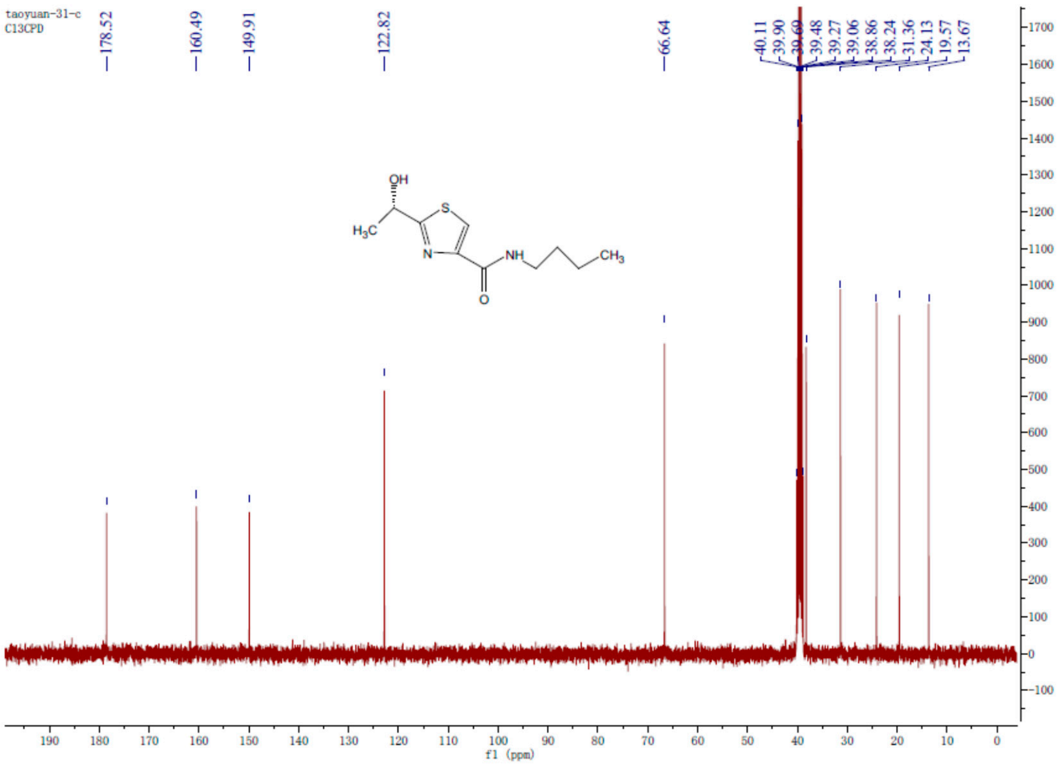

Figure S6. ^{13}C NMR of Compound **10a** (*d*$_6$-DMSO).

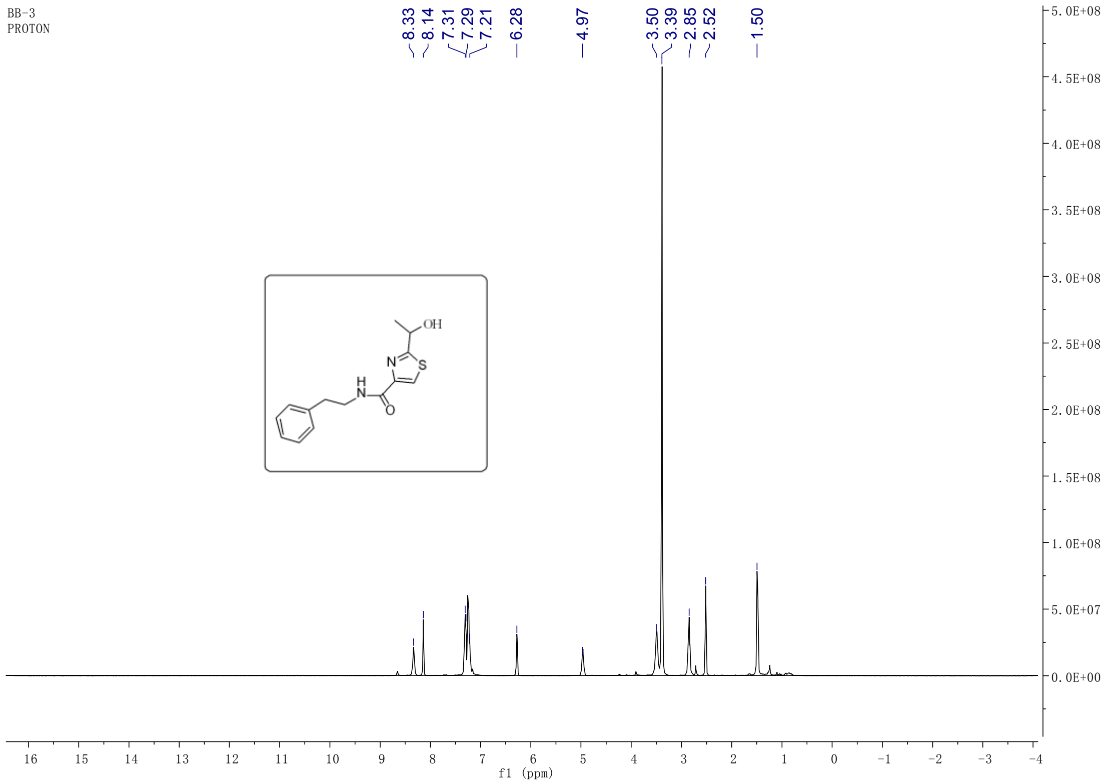

Figure S7. ^1H NMR of Compound **10b** (*d_6*-DMSO).

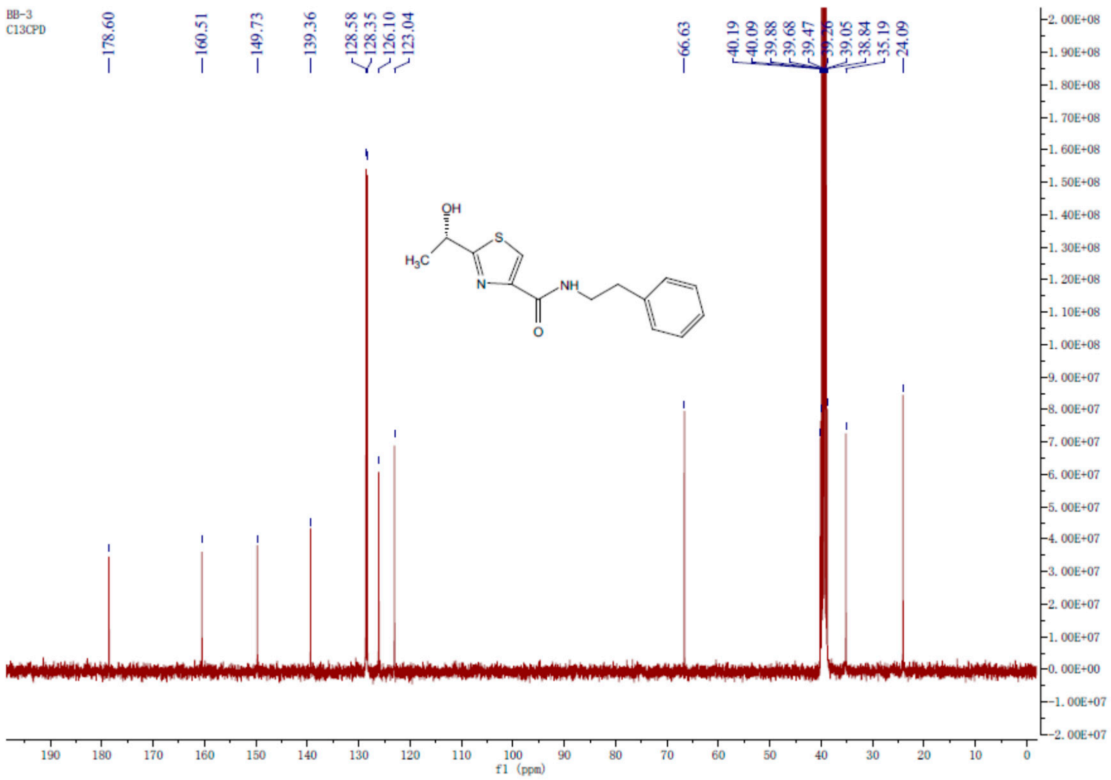

Figure S8. ^{13}C NMR of Compound **10b** (*d_6*-DMSO).

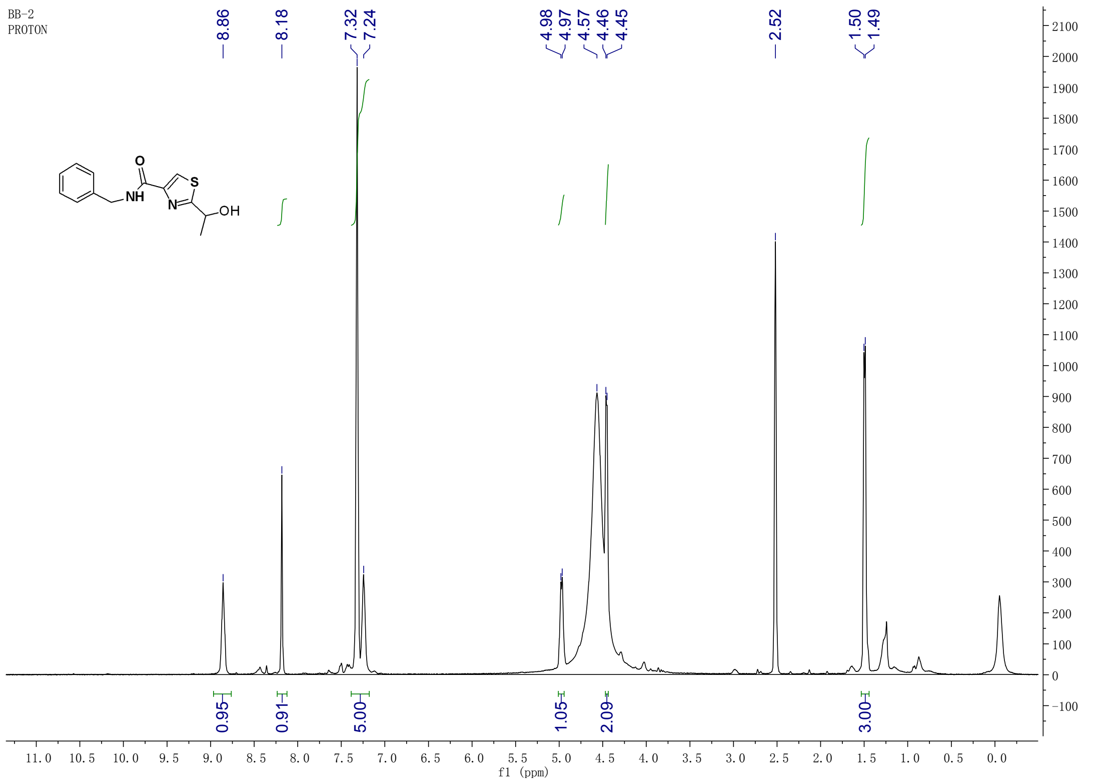

Figure S9. ^{1}H NMR of Compound **10c** (d_6-DMSO).

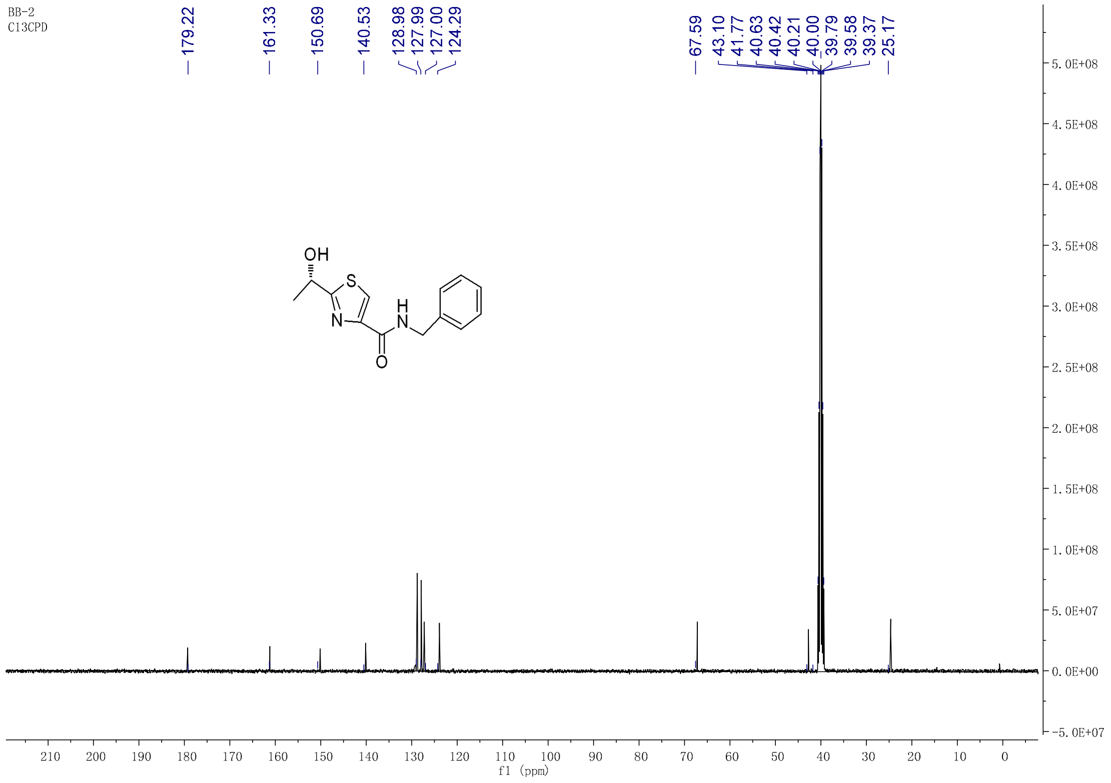

Figure S10. ^{13}C NMR of Compound **10c** (d_6-DMSO).

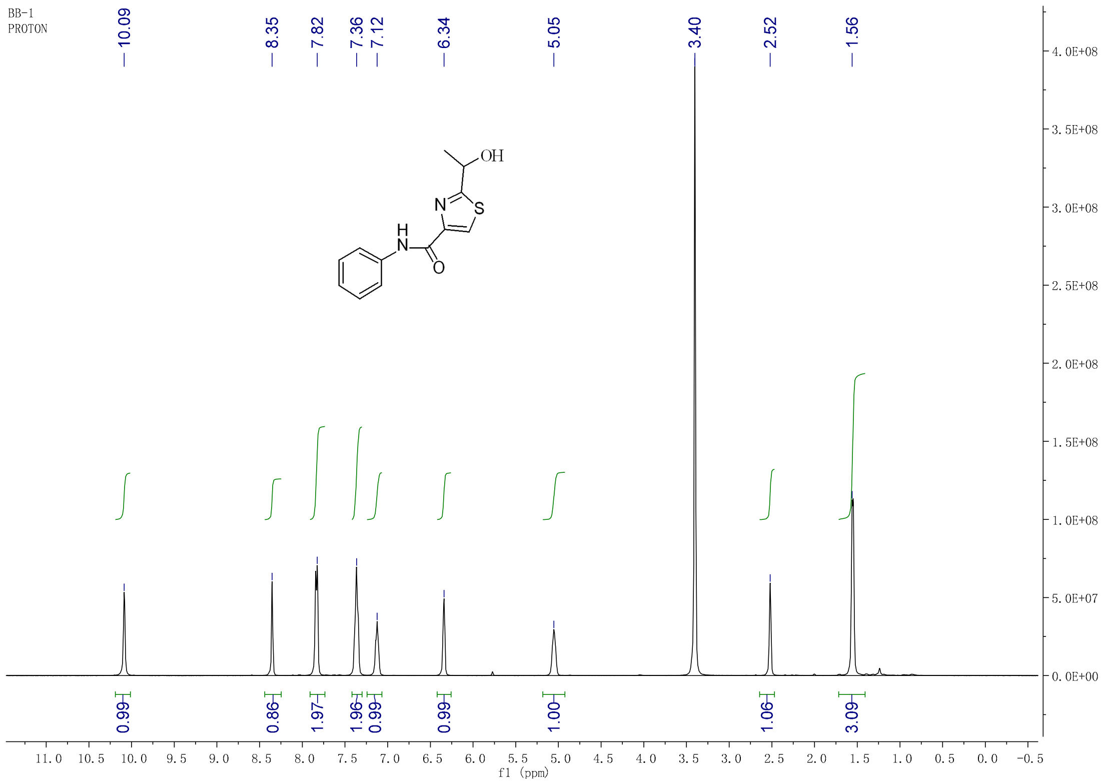

Figure S11. ^{1}H NMR of Compound **10d** (d_6-DMSO).

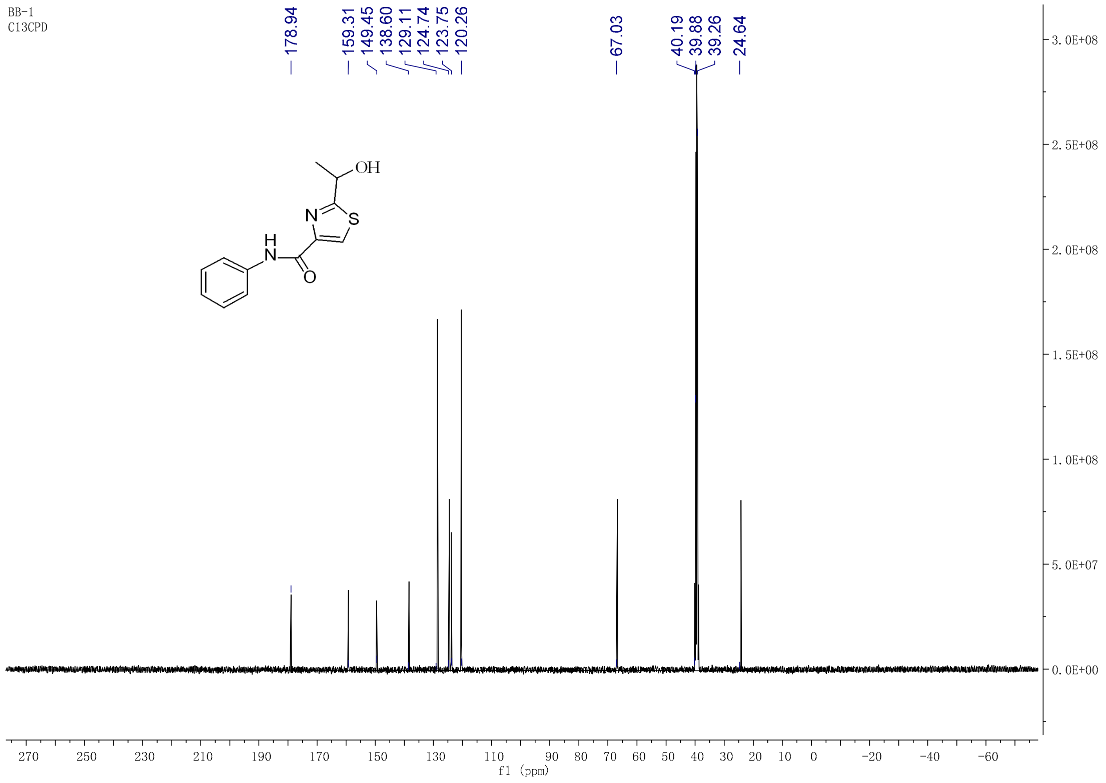

Figure S12. ^{13}C NMR of Compound **10d** (d_6-DMSO).
